# A High-Efficiency Deep Blue Emitter for OLEDs with a New Dual-Core Structure Incorporating ETL Characteristics

**DOI:** 10.3390/molecules28227485

**Published:** 2023-11-08

**Authors:** Yeongjae Heo, Hyukmin Kwon, Sangwook Park, Sunwoo Dae, Hayoon Lee, Kiho Lee, Jongwook Park

**Affiliations:** Integrated Engineering, Department of Chemical Engineering, Kyung Hee University, Gyeonggi 17104, Republic of Korea; youngxi_0514@khu.ac.kr (Y.H.); hm531@khu.ac.kr (H.K.); pswook@khu.ac.kr (S.P.); sunwoo4674@khu.ac.kr (S.D.); kssarang1@khu.ac.kr (H.L.); kiholee@khu.ac.kr (K.L.)

**Keywords:** OLED, blue, dual core, side group, oxazole, ETL

## Abstract

In this study, we introduced the weak electron-accepting oxazole derivative 4,5-diphenyl-2-(4-(4,4,5,5-tetramethyl-1,3,2-dioxaborolan-2-yl)phenyl)oxazole (TPO) into both anthracene and pyrene moieties of a dual core structure. Ultimately, we developed 2-(4-(6-(anthracen-9-yl)pyren-1-yl)phenyl)-4,5-diphenyloxazole (AP-TPO) as the substitution on the second core, pyrene, and 4,5-diphenyl-2-(4-(10-(pyren-1-yl)anthracen-9-yl)phenyl)oxazole (TPO-AP) as the substitution on the first core, anthracene. Both materials exhibited maximum photoluminescence wavelengths at 433 and 443 nm in solution and emitted deep blue light with high photoluminescence quantum yields of 82% and 88%, respectively. When used as the emitting layer in non-doped devices, TPO-AP outperformed AP-TPO, achieving a current efficiency of 5.49 cd/A and an external quantum efficiency of 4.26% in electroluminescence. These materials introduce a new category of deep blue emitters in the organic light-emitting diodes field, combining characteristics related to the electron transport layer.

## 1. Introduction

**Figure 1:**
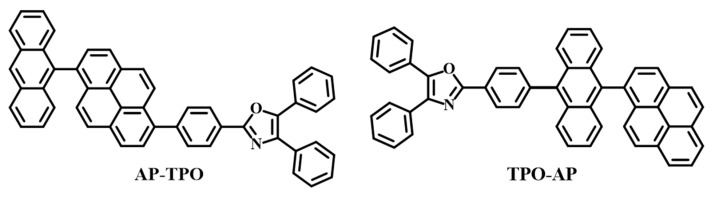
Chemical structures of AP-TPO and TPO-AP.

The conventional blue-emitting materials for organic light-emitting diodes (OLEDs) have primarily used single-core chromophores with an excellent photoluminescence quantum yield (PLQY), such as anthracene, pyrene, and fluorene [1,2,3]. However, these chromophores are planar, which increases the intermolecular packing in the film state and promotes excimer formation, leading to reduced electroluminescence (EL) efficiency and color purity [4,5,6]. Also, bulky side groups are typically added to the chromophore to prevent molecular interaction. However, in our study, rather than using side groups, we combined anthracene and pyrene to create a dual-core chromophore, the AP-Core chromophore, maintaining a dihedral angle of approximately 90 degrees between the two moieties. This strategy aimed to prevent excimer formation and suppress intermolecular packing, leading to the development of a new chromophore. Our approach resulted in more than twice the efficiency and device lifetime compared to materials using anthracene and pyrene as single cores [7]. Subsequent research introduced various side groups, symmetrically or asymmetrically, based on the dual core to provide blue-emitting materials [8]. However, in this study, we incorporated the same side group on each of the dual cores, anthracene and pyrene, using the 4,5-diphenyl-2-(4-(4,4,5,5-tetramethyl-1,3,2-dioxaborolan-2-yl)phenyl)oxazole (TPO) moiety. TPO possesses a weak electron-accepting characteristic that was not previously applied to the AP-Core, aiming to enhance charge balance [9,10]. This molecular design allowed for a detailed examination of the roles of the first core, anthracene, and the second core, pyrene. Finally, we synthesized two new blue-emitting materials, 2-(4-(6-(anthracen-9-yl)pyren-1-yl)phenyl)-4,5-diphenyloxazole (AP-TPO) and 4,5-diphenyl-2-(4-(10-(pyren-1-yl)anthracen-9-yl)phenyl)oxazole (TPO-AP). We also investigated the changes in the photophysical, thermal, and EL properties of the synthesized compounds (Figure 1).

## 2. Results and Discussion

### 2.1. Photophysical Properties and Theoretical Calculations

**Figure 2:**
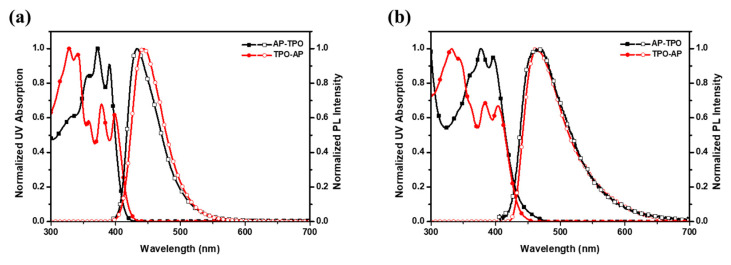
Normalized UV-visible absorption and PL spectra of newly synthesized compounds: (**a**) in solution state (concentration: 1 × 10^−5^ M in toluene), (**b**) in evaporated film state (thickness: 50 nm).

We prepared TPO-AP and AP-TPO by substituting side groups on the first core, anthracene, and the second core, pyrene, based on the AP-Core. The AP-Core maintains orthogonality, preventing the extension of molecular conjugation, making it a suitable core for a blue-emitting material. The TPO exhibits a weak electron-accepting characteristic which can enhance the overall charge balance of the molecule. Table 1 and Figure 2 provide a comprehensive summary of the ultraviolet-visible (UV-Vis) absorption and the photoluminescence (PL) spectra of the synthesized compounds in both the solution and film states. In the solution state, the AP-TPO shows main absorption peaks at 371 nm and 390 nm, while the TPO-AP shows main absorption peaks at 328 nm and 341 nm. Both compounds share a common absorption attributed to the TPO in the 300–340 nm range, and the absorption in the 340–400 nm range is due to the π-π* transition of the anthracene and pyrene cores [9,11,12]. However, the AP-TPO shows a high absorption in the AP-Core region, and the TPO-AP exhibits a high absorption in the TPO absorption region. 

**Figure 3:**
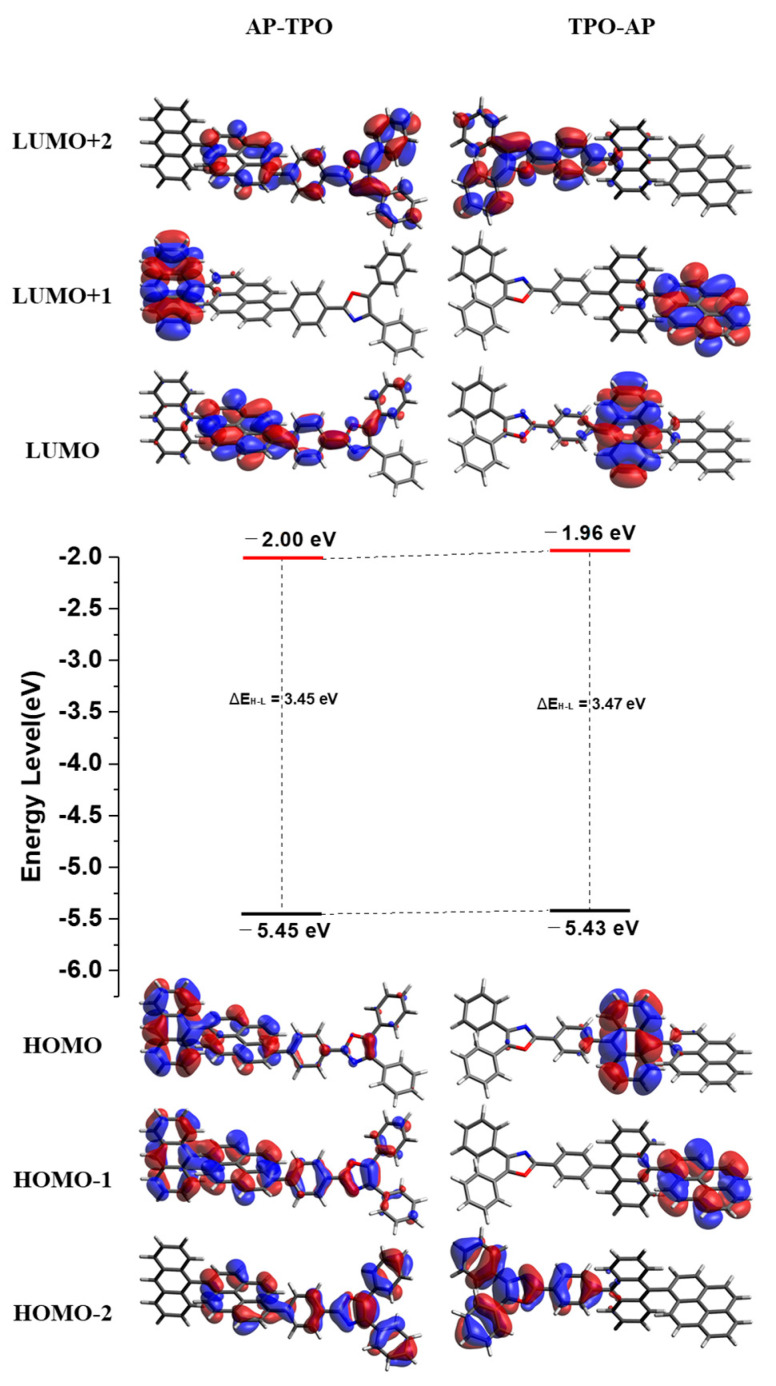
Electron density distributions of HOMO−2, HOMO−1, HOMO, LUMO, LUMO+1, and LUMO+2 calculated with B3LYP/6-311G(d).

Density functional theory (DFT) and time-dependent DFT (TD-DFT) calculations were performed using the B3LYP/6-311G(d) method and basis set with the ORCA program [13]. We calculated the optimized structures of the synthesized materials, as well as the energy level values at highest occupied molecular orbital (HOMO), the lowest unoccupied molecular orbital (LUMO), and the frontier orbitals using DFT (Figure 3). Furthermore, we used the TD-DFT to compute the expected absorption wavelengths, the oscillator strengths at those wavelengths, the transition states, and the contributions at each transition state for each material (Table 2). In previous studies, it was noted that in the AP-Core, the electron density distribution primarily resides in anthracene for the HOMO and LUMO, while HOMO−1 and LUMO+1 are mainly distributed in pyrene. Based on this, anthracene was denoted as the first core and pyrene as the second core. Additionally, previous studies indicated that the transition with the highest oscillator strength occurs between HOMO and LUMO at the longest wavelength [8]. However, AP-TPO and TPO-AP exhibited different trends. In the case of the AP-TPO, the electron density is evenly spread across the molecule in HOMO and HOMO−1, and in LUMO, it is predominantly distributed in pyrene and the TPO side. At the wavelength of the highest oscillator strength, 386 nm, the most significant contribution was observed in the transition from HOMO−1 to LUMO, accounting for 55%. On the other hand, the transition from HOMO to LUMO was calculated to be 21%. For the TPO-AP, in both HOMO and LUMO, the electron density is primarily present in anthracene, and in LUMO, it is partially present in the TPO. HOMO−1 and LUMO+1 mainly have electron density in pyrene, exhibiting a trend similar to previous AP-core derivatives. However, at the wavelength where the oscillator strength is the highest, 382 nm, the transition from HOMO−2 to LUMO+2, with an 83% contribution, was identified as the primary transition. The electron density of HOMO−2 and LUMO+2 in the TPO-AP is mainly distributed in the TPO moiety. Consequently, considering the major transition with a significant contribution at the wavelength with the highest oscillator strength, it can be presumed that absorption occurs predominantly in the AP-Core for the AP-TPO and in the TPO moiety for the TPO-AP. In the film state, the AP-TPO exhibited maximum absorption at 377 nm and 396 nm, while the TPO-AP exhibited maximum absorption at 332 nm and 345 nm, with trends similar to those in the solution state. However, transitioning from the solution state to the film state resulted in molecular interactions due to the close proximity of molecules, causing a red-shift and broadening of the UV-Vis and PL spectra. When compared with the solution state, the absorption wavelengths of the AP-TPO and the TPO-AP red-shifted by 6 nm and 4 nm, respectively, in the film state, and they showed a broader shape [14]. 

**Figure 4:**
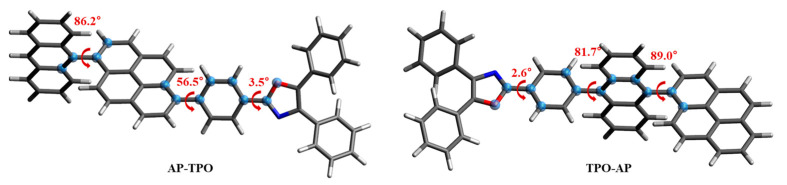
Optimized structures of AP-TPO and TPO-AP.

In the solution state, the PL maximum (PLmax) values for the AP-TPO and the TPO-AP were 433 nm and 443 nm, respectively, indicating emission of light in the deep blue region for both materials. In the film state, the PLmax values for the AP-TPO and the TPO-AP were 464 nm and 462 nm, respectively, signifying a bathochromic shift of 32 nm and 19 nm, respectively, compared to the solution state. Moreover, the full width at the half maximum (FWHM) values were 80 nm and 72 nm for AP-TPO and TPO-AP, respectively, in the film state, representing an increase of 26 nm and 17 nm, respectively, compared to the solution state. The significant difference and broader FWHM in the PLmax between the solution and film states of AP-TPO are believed to result from stronger molecular interactions and packing compared to TPO-AP [15]. A detailed examination of the optimized structures using molecular calculations revealed that the dihedral angle of the AP-Core exhibited the anticipated orthogonal structure. However, in the AP-TPO, the dihedral angle between the pyrene and the TPO was 56.5°, and in the TPO-AP, the dihedral angle between the anthracene and the TPO was approximately 81.7°, which is close to 90°. Therefore, it is presumed that the TPO-AP, which effectively prevents intermolecular packing, contributes to the change in PLmax. Both synthesized materials exhibited an emission in the blue region in the film state (Figure 4).

In both the solution and film states, the AP-TPO exhibited a PLQY of 82% and 20%, while the TPO-AP showed values of 88% and 24%. Generally, luminescence efficiency decreases in the film state compared to the solution state due to numerous non-radiative pathways resulting from closer intermolecular distances, which leads to quenching [16]. However, the TPO-AP demonstrated a higher PLQY compared to the AP-TPO due to the substitution of pyrene as the second core and the TPO as the side group on both sides of the anthracene, resulting in steric hindrance and a highly twisted structure. This hindered intermolecular packing and yielded a higher PLQY. These increased PLQY values are beneficial for achieving an increased EL efficiency in OLED devices. The time-resolved photoluminescence (TRPL) was used to measure fluorescence lifetime (τ_F_) which allowed for the calculation of the radiative rate constant (*k_r_*) and the non-radiative rate constant (*k_nr_*). Time decay curves of these compounds showed a mono-exponential type and a prompt fluorescence emission with no delayed fluorescence component (Figure S1) [17]. The τ_F_ values for the AP-TPO and the TPO-AP were measured as 1.26 and 1.36 ns, respectively. The AP-TPO and the TPO-AP both exhibited similar values for *k_r_* at 6.50 and 6.48 × 10^8/^s, respectively. However, the AP-TPO had a higher *k_nr_* of 14.3 × 10^7^/s compared to the TPO-AP’s 8.83 × 10^7^/s, resulting in a lower PLQY.

### 2.2. Thermal and Electrical Properties

**Figure 5:**
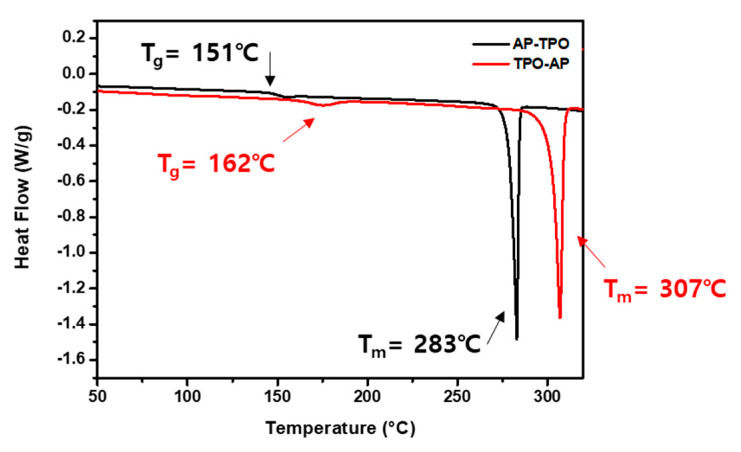
DSC curves of the synthesized AP-TPO and TPO-AP.

The thermal properties of the synthesized materials were determined using thermogravimetric analyzers (TGA) and differential scanning calorimetry (DSC). The results showed that the AP-TPO had values of 448 °C, 151 °C, and 283 °C for degradation temperatures (T_d_, 5% weight loss temperature), glass-transition temperature (T_g_), and melting temperature (T_m_), respectively. The TPO-AP exhibited measurements of 395 °C, 162 °C, and 307 °C for T_d_, T_g_, and T_m_, respectively (Table 3 and Figure 5). The incorporation of the TPO side group demonstrated superior thermal stability compared to the AP-Core. The AP-TPO exhibited a higher T_d_ compared to the TPO-AP. This can be attributed to the highly twisted conformation between the anthracene and the TPO in the TPO-AP, leading to an increased internal molecular instability and, consequently, a lower T_d_ when compared to the AP-TPO. However, the T_g_ of the TPO-AP is higher than that of the AP-TPO. Additionally, all the thermal properties of both materials are well above the temperatures required for stable device operation, making them suitable for use in device processing and operation [18]. Moreover, the measurements were conducted for device preparation, determining the HOMO level, band gap, and the LUMO level. The HOMO energy level was determined using AC-2, and the LUMO energy level was calculated by adding the band gap energy to the measured HOMO energy level. The band gap energy was estimated from the absorption edge in the thin film state using a plot of (*hv*) vs. (*αhv*)^2^, where *α*, *h*, and *v* represented the absorbance, Planck’s constant, and frequency of light, respectively. The measured HOMO levels for the AP-TPO and the TPO-AP were −5.80 eV and −5.84 eV, respectively, showing similarity. The measured band gaps were also similar, with the AP-TPO at 2.92 eV and the TPO-AP at 2.88 eV, with the longer conjugation length of the TPO-AP resulting in a slightly smaller band gap. To aid in understanding the experimental data of the synthesized materials, we performed theoretical calculations to obtain the trend of relative changes by comparing the theoretical values. Generally, the DFT calculation methods commonly used for the computation of the HOMO and LUMO energy levels are primarily designed for total energy calculations and the description of optimized structures. They are not oriented towards achieving precise molecular orbital calculations which can lead to discrepancies between the calculated and measured values [19,20]. To facilitate the relative comparison between theoretical predictions and experimental measurements, we conducted molecular calculations to determine the HOMO and LUMO energy levels. The confirmed HOMO levels for the AP-TPO and the TPO-AP were −5.45 eV and −5.42 eV, respectively, while the LUMO levels were −2.00 eV and −1.95 eV, respectively. These values may differ from the experimental measurements, but the trends were found to be similar.

### 2.3. Electroluminescence Properties

**Figure 6:**
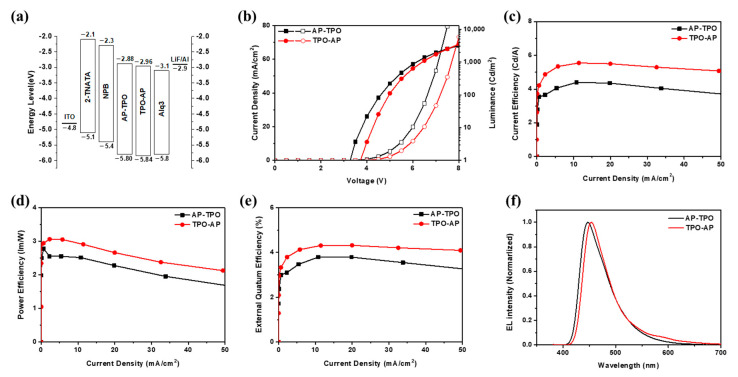
EL performances of AP-TPO and TPO-AP: (**a**) energy levels of the fabricated devices, (**b**) J-V-L curve, (**c**) CE curves, (**d**) PE curves, (**e**) EQE curves, and (**f**) EL spectra.

Using the measured energy levels, non-doped OLED devices were fabricated with AP-TPO and TPO-AP as the EML using the following structure: ITO/2-TNATA (60 nm)/NPB (15 nm)/emissive layer (EML) (35 nm)/Alq_3_ (20 nm)/LiF (1 nm)/Al (200 nm). The properties of the OLED devices at 10 mA/cm^2^ are summarized in Table 4. The operating voltages for the fabricated AP-TPO and TPO-AP devices were measured at 5.44 V and 5.86 V at 10 mA/cm^2^, respectively. The two materials, having similar molecular structures and energy levels, exhibited comparable operating voltages [21,22] (Figure 6a,b). The turn-on voltage of the two devices, AP-TPO and TPO-AP, was observed to be relatively higher at 3.27 V and 3.73 V at 1 cd/m^2^, respectively, compared to the commercialized blue devices. In particular, the TPO-AP exhibited a slightly higher turn-on voltage. This is believed to be attributed to the interface properties between the hole transporting layer (HTL) of the NPB and the EML. The interface characteristics appear to be fine for the AP-TPO but relatively poorer for the TPO-AP. Further investigations involving dielectric constant and contact angle studies of the NPB material and both materials will be discussed in future research. The power efficiency (PE) for both devices was similar, with values of 2.52 lm/W for the AP-TPO and 2.94 lm/W for the TPO-AP (Figure 6d). However, the current efficiency (CE) for the AP-TPO and the TPO-AP was 4.33 cd/A and 5.49 cd/A, respectively, and the external quantum efficiency (EQE) was 3.73% for the AP-TPO and 4.26% for the TPO-AP, indicating that the TPO-AP exhibited a higher CE than the AP-TPO (Figure 6c,e). This is attributed to the TPO substituents on the first core, anthracene, effectively suppressing intermolecular packing, leading to a high PLQY. Furthermore, the measurement of transient EL revealed delayed fluorescence in both synthesized materials (Figure S2). This delayed fluorescence is attributed to the triplet-triplet annihilation (TTA) occurring in the anthracene and the pyrene moieties during the operation of the EL device. It is known that anthracene and pyrene are moieties where the TTA occurs efficiently during the operation of the EL devices [23,24]. When measuring the LTPL of the two synthesized materials, the triplet energy level (T_1_) calculated from the band edge of each spectrum for the AP-TPO and the TPO-AP was found to be 2.61 and 2.23 eV, respectively (Figure S3). The singlet energy level (S_1_) was determined to be 3.05 eV for the AP-TPO and 3.01 eV for the TPO-AP through measurement of PL at room temperature. Due to the longer molecular conjugation in the TPO-AP, its S_1_ and T_1_ values were lower than those of the AP-TPO. Both synthesized materials exhibited significant ΔE_ST_ values of 0.44 and 0.78 eV, indicating the difficulty of the thermally activated delayed fluorescence process. The TPO-AP, with a higher occurrence of the TTA, showed a longer transient EL response compared to the AP-TPO, which is believed to contribute to its higher EQE [25]. Generally, polycyclic aromatic hydrocarbons tend to exhibit hole-dominant properties with lower electron mobility, causing an imbalance in the charge within the device and leading to an efficiency roll-off [26,27]. However, the two materials synthesized in this study incorporate the TPO, which has weak electron-accepting characteristics, into the AP-core, improving the overall carrier balance and minimizing the roll-off, even at higher current densities. The EL maximum (ELmax) for the AP-TPO and the TPO-AP were observed at 447 nm and 453 nm, respectively, with CIE coordinates of (0.156, 0.134) and (0.168, 0.154), respectively, indicating blue emission (Figure 6f). The ELmax of the AP-TPO showed an approximately 17 nm blue shift compared to the film PL max, while the TPO-AP exhibited a 9 nm hypsochromic shift. However, the shape of the PL and the EL spectra was similar. This can be attributed to the recombination zone (RZ) existing within the EML due to the fast hole mobility, resulting in its formation closer to the ETL. To address this, we aim to adjust the thickness of the HTL and ETL layers or consider the substitution of a high electron mobility ETL in future research [28]. The device lifetime (LT) of the AP-TPO and the TPO-AP was measured at 1000 nits, and the LT_50_ for each device was found to be 0.54 and 3.1 h, respectively (Figure S4). In the case of the TPO-AP, where the side groups are substituted on both sides of the first core, anthracene, it is believed that this molecular design efficiently hinders the intermolecular packing, reduces the non-radiative pathways, and enables stable device operation, resulting in the longer lifetime observed in the TPO-AP.

## 3. Experimental

### 3.1. Materials, Measurements, and Device Fabrication

The synthesized compounds were confirmed using a Bruker Avance 400 for proton NMR spectroscopy (Bruker, Billerica, MA, USA). The optical properties were assessed using an HP 8453 UV-VISNIR spectrometer (Agilent, Santa Clara, CA, USA) for UV-Vis absorption and a Perkin-Elmer luminescence spectrometer LS50 (Perkin-Elmer, Waltham, MA, USA) with a Xenon flash tube for PL. The T_d_ of materials were determined using TGA (SDT Q600, TA Instruments, New Castle, DE, USA). The T_g_ and T_m_ of the compounds were determined using DSC under a nitrogen atmosphere with a DSC4000 (PerkinElmer, MA, USA). The HOMO energy level was measured by a photoelectron spectrometer (AC-2, Riken Keiki, Tokyo, Japan). The low temperature PL (LTPL) was obtained by a JASCO FP-8500 spectrofluorometer (JASCO, Tokyo, Japan). The transient EL was carried out at 10 mA/cm^2^. The aspect ratio of the pulse applied from the 33600A function generator (KEYSIGHT, Santa Rosa, CA, USA) is 1:1, and the pulse width is 500 us. The TRPL curves were obtained by a quantaurus-tau fluorescence lifetime spectrometer C11367-11 (HAMAMATSU PHOTONICS K.K, Hamamatsu, Japan). Non-doped OLED devices for blue emission were fabricated with the following structure: ITO/2-TNATA (60 nm)/NPB (15 nm)/EML (35 nm)/Alq_3_ (20 nm)/LiF (1 nm)/Al (200 nm). In this structure, the 2-TNATA represents 4,4′,4″-*tris*(*N*-(2-naphthyl)-*N*-phenylamino)-triphenylamine and serves as the hole injection layer (HIL); the *N*,*N*′-bis(naphthalen-1-yl)-*N*,*N*′-bis(phenyl)benzidine (NPB) functions as the HTL; the Tris(8-hydroxyquinolinato)aluminium (Alq_3_) forms the electron transporting layer (ETL); the electron injection layer (EIL) consists of lithium fluoride (LiF); and the ITO is used as the anode, while the aluminum (Al) serves as the cathode. The organic compounds were thermally evaporated at a vacuum pressure of 10^−6^ torr and formed an emitting layer with an area of 4 mm^2^ at a deposition rate of 0.1 nm/s. The aluminum layer was also deposited under a pressure of 10^−6^ torr. The current density-voltage (J-V) characteristics of the fabricated OLED devices were evaluated using a Keithley 2400 Source Meter (Keithley, Cleveland, OH, USA). The EL spectrum of the devices was measured with a Minolta CS-1000A spectroradiometer (Konica Minolta, Tokyo, Japan).

### 3.2. Synthesis

**Scheme 1:**
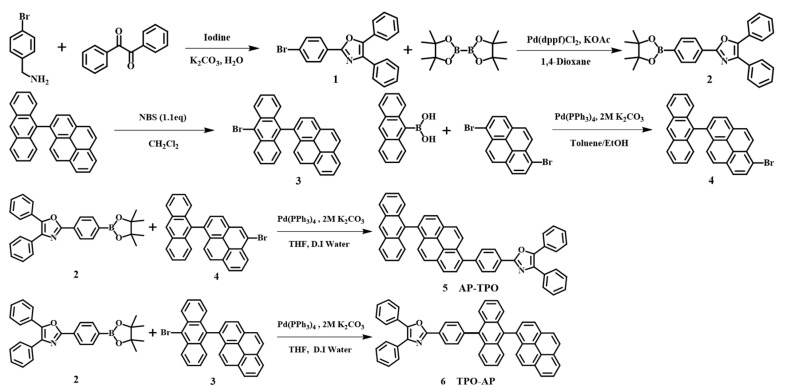
Synthetic routes of AP-TPO and TPO-AP.

The synthesis method for compound (**4**) was previously described in a prior paper [7]. Scheme 1 illustrates the synthetic pathways for AP-TPO and TPO-AP.

#### 3.2.1. 2-(4-Bromophenyl)-4,5-diphenyloxazole (**1**)

(4-Bromophenyl)methanamine (1.00 g, 5.37 mmol), benzyl (1.13 g, 5.37 mmol), potassium carbonate (K_2_CO_3_) (2.23 g, 16.12 mmol), and iodine (0.41 g, 1.61 mmol) were added to a 250 mL three-necked flask. 50 mL of water was placed in a round-bottom flask, and the mixture was stirred at 60 °C for 8 h. After the reaction was complete, the mixture was extracted with ethyl acetate (EA) and distilled water (DI water). The organic layer was dried with anhydrous magnesium sulfate anhydrous (MgSO_4_) and then filtered. The solution was evaporated, and then it was purified by column chromatography using EA and hexane in a 1:19 ratio as the eluent. (Yield: 15%) ^1^H NMR (400 MHz, CDCl_3_, δ) 8.03–7.99 (d, 2H), 7.72–7.61 (m, 6H), 7.41–7.36 (m, 6H).

#### 3.2.2. 4,5-Diphenyl-2-(4-(4,4,5,5-tetramethyl-1,3,2-dioxaborolan-2-yl)phenyl)oxazole (**2**)

Compound (**1**) (0.50 g, 1.33 mmol), bis(pinacolato)diboron (0.57 g, 2.26 mmol), potassium acetate (KOAc) (0.39 g, 3.99 mmol), and bis(diphenylphosphino)ferrocene)palladium(II) dichloride (Pd(dppf)Cl_2_) (0.05 g, 0.07 mmol) were added into a 100 mL three-necked flask under a nitrogen atmosphere. 25 mL of 1,4-dioxane was also added, and the mixture was refluxed overnight at 60 °C. After the reaction was complete, the mixture was extracted with EA and DI water. The organic layer was dried with anhydrous MgSO_4_ and then filtered. The solution was evaporated, and it was then purified by column chromatography using EA and hexane in a 1:19 ratio as the eluent. (Yield: 74%) ^1^H NMR (400 MHz, CDCl_3_, δ) 8.16–8.12 (d, 2H), 7.93–7.89 (d, 2H), 7.73–7.67 (m, 4H), 7.42–7.34 (m, 6H), 1.38–1.35 (s, 12H).

#### 3.2.3. 1-(10-Bromoanthracen-9-yl)pyrene (**3**)

1-(Anthracen-9-yl)pyrene (0.20 g, 0.53 mmol) and N-bromosuccinimide (0.10 g, 0.58 mmol) were added to 10 mL of methylene chloride (MC). Acetic acid (1.0 mL) was then added into the reaction. The mixture was stirred at 25 °C for 3 h. After the reaction was complete, the reaction mixture was extracted with MC and DI water. The organic layer was dried with anhydrous MgSO_4_ and then filtered. The solution was evaporated. Then, it was purified by column chromatography using hexane and toluene in a 9:1 ratio as the eluent. The precipitate was filtered, washed with ethanol, and a yellow compound was obtained (Yield: 76%). ^1^H NMR (400 MHz, CDCl_3_, δ) 8.61–8.57 (d, 2H), 8.54–8.51 (d, 1H), 8.39–8.34 (m, 2H), 8.33–8.30 (d, 1H), 8.24–8.20 (d, 1H), 8.11–8.04 (m, 2H), 7.92–7.89 (d, 1H), 7.74–7.68 (q, 2H), 7.38–7.32 (q, 2H), 7.25–7.20 (d, 2H), 7.11–7.06 (m, 1H).

#### 3.2.4. 2-(4-(6-(Anthracen-9-yl)pyren-1-yl)phenyl)-4,5-diphenyloxazole (**5**) (AP-TPO)

Compound (**2**) (0.36 g, 0.85 mmol), Compound (**4**) (0.30 g, 0.66 mmol), palladium-tetrakis(triphenylphosphine) (Pd(PPh_3_)_4_) (0.023 g, 0.02 mmol), and K_2_CO_3_ (0.27 g, 1.97 mmol) were added to a 100 mL three-necked flask. Anhydrous THF (15 mL) was added, followed by the consecutive addition of DI water (3 mL). The mixture was then refluxed and stirred under nitrogen at 80 °C for 14 h. After the reaction was complete, it was extracted with MC and DI water. The organic layer was dried with anhydrous MgSO_4_ and then filtered. The solution was evaporated. It was then purified by column chromatography using a 3:7 ratio of hexane and MC as the eluent. (Yield: 57%) ^1^H NMR (400 MHz, DMSO, δ) 8.86–8.82 (s, 1H), 8.55–8.51 (d, 1H), 8.39–8.23 (m, 7H), 8.13–8.06 (q, 2H), 7.98–7.94 (d, 1H), 7.90–7.85 (d, 2H), 7.75–7.68 (m, 4H), 7.55–7.40 (m, 8H), 7.31–7.26 (t, 2H), 7.21–7.12 (q, 3H). ^13^C NMR (101 MHz, THF-d_8_) δ 159.95, 145.83, 143.45, 137.13, 137.00, 135.16, 134.35, 132.84, 131.74, 131.35, 131.16, 131.10, 131.00, 129.62, 129.25, 128.73, 128.51, 128.40, 128.10, 127.94, 127.86, 127.75, 127.72, 127.14, 126.77, 126.73, 126.57, 126.40, 125.73, 125.64, 125.24, 125.21, 125.15, 125.07, 124.97, 124.82. *m*/*z* = 674.25; calcd. For C51H51NO: 673.24 [M+] Anal. calcd. for C51H51NO: C, 90.91; H, 4.64; N, 2.08; O, 2.37% found: C, 90.89; H, 4.71; N, 2.08; O, 2.21%.

#### 3.2.5. 4,5-Diphenyl-2-(4-(10-(pyren-1-yl)anthracen-9-yl)phenyl)oxazole (**6**) (TPO-AP)

Compound (**2**) (0.36 g, 0.85 mmol), Compound (**3**) (0.30 g, 0.66 mmol), Pd(PPh_3_)_4_ (0.023 g, 0.02 mmol), and K_2_CO_3_ (0.27 g, 1.97 mmol) were added to a 100 mL three-necked flask. Anhydrous THF (15 mL) was added, followed by the consecutive addition of DI water (3 mL). The mixture was then refluxed and stirred under nitrogen at 80 °C for 14 h. After the reaction was complete, the mixture was extracted with MC and DI water. The organic layer was dried with anhydrous MgSO_4_ and then filtered. The solution was evaporated. It was then purified by column chromatography using a 1:1 ratio of hexane and MC as the eluent. (Yield: 77%) ^1^H NMR (400 MHz, THF, δ) 8.49–8.43 (m, 3H), 8.29–8.21 (q, 3H), 8.17–8.13 (d, 1H), 8.07–7.99 (m, 2H), 7.86–7.70 (m, 9H), 7.46–4.30 (m, 11H), 7.21–7.16 (t, 2H). ^13^C NMR (101 MHz, THF-d_8_) δ 159.98, 145.88, 141.60, 137.01, 136.68, 135.84, 134.08, 132.88, 131.97, 129.32, 128.66, 128.31, 128.02, 127.89, 127.36, 127.06, 126.79, 126.61, 126.48, 125.31. *m*/*z* = 674.25; calcd. For C51H51NO: 673.24 [M+] Anal. calcd. for C51H51NO: C, 90.91; H, 4.64; N, 2.08; O, 2.37% found: C, 90.27; H, 4.51; N, 2.09; O, 2.91%. 

## 4. Conclusions

The AP-Core based molecules, AP-TPO and TPO-AP, were synthesized by substituting the weak electron-accepting TPO as the second core and the first core, respectively. In the solution state, both the AP-TPO and the TPO-AP exhibited PL maxima at 433 nm and 443 nm, respectively, with high PLQYs of 82% and 88%, respectively, indicating efficient luminescence. When applied as the EML in OLED devices, the AP-TPO and the TPO-AP demonstrated CE values of 4.33 cd/A and 5.49 cd/A, respectively, as well as EQE values of 3.73% and 4.26%, respectively. As a result, the TPO-AP showed a higher efficiency in OLED devices. The introduction of side groups on the first core, anthracene, effectively suppresses the intermolecular packing, allowing for high efficiency. Moreover, the incorporation of oxazole side groups helps maintain a charge balance even at high current densities. These results emphasize the potential of AP-TPO and TPO-AP for application in high-efficiency OLED devices.

## Figures and Tables

**Table 1:** Photophysical properties of synthesized compounds.

	Solution ^a^			Film ^b^			PLQY ^c^ (%)
	UV_max_ (nm)	PL_max_ (nm)	FWHM (nm)	UV_max_ (nm)	PL_max_ (nm)	FWHM (nm)	
AP-TPO	371, 390	433	54	377, 396	464	80	82/20
TPO-AP	328, 341	443	58	332, 345	462	72	88/24

^a^ Toluene solution (1.0 × 10^−5^ M). ^b^ Film thickness: 50 nm on a glass substrate. ^c^ Absolute PLQY of solution state/film state.

**Table 2:** Absorption frequencies and oscillator strengths calculated with B3LYP/6-311G(d) for AP-TPO and TPO-AP.

Compound	Absorption Wavelength (nm)	Oscillator Strength	Characteristic of Transition	Contribution ^a^ (%)
AP-TPO	412.9	0.011	HOMO−1→ LUMO HOMO → LUMO	23.5 70.0
	403.2	0.014	HOMO−1→ LUMO+1 HOMO → LUMO+1	52.7 34.7
	386.1	1.321	HOMO−1→ LUMO HOMO → LUMO+1 HOMO → LUMO	54.5 12.8 21.6
	363.9	0.010	HOMO−1→ LUMO+1 HOMO → LUMO+1	25.1 51.0
TPO-AP	399.4	0.005	HOMO → LUMO	93.4
	398.7	0.003	HOMO−1→ LUMO+1	94.0
	382.8	0.383	HOMO−2→ LUMO+2	83.9
	372.7	0.164	HOMO−1→ LUMO HOMO → LUMO+1	19.1 78.7

^a^ When the sum of contributions is less than 100%, the remaining contributions include various small portions of transitions. Only the main process was indicated.

**Table 3:** Thermal and electrical properties of AP-TPO and TPO-AP.

	T_d_ (°C)	T_g_ (°C)	T_m_ (°C)	HOMO (eV)	LUMO (eV)	Band Gap (eV)
AP-TPO	448	151	283	−5.80	−2.88	2.92
TPO-AP	395	162	307	−5.84	−2.96	2.88

**Table 4:** EL performances of non-doped OLED devices at 10 mA/cm^2^.

	Volt (V)	CE (Cd/A)	PE (lm/W)	EQE (%)	CIE (x, y)	EL_max_ (nm)
AP-TPO	5.44	4.33	2.52	3.73	(0.156, 0.134)	447
TPO-AP	5.86	5.49	2.94	4.26	(0.168, 0.154)	453

## Data Availability

Data are contained within the article and Supplementary Materials.

## Supplementary Materials

The following supporting information can be downloaded at: https://www.mdpi.com/article/10.3390/molecules28227485/s1, Figure S1: TRPL curves of AP-TPO and TPO-AP; Figure S2: Transient EL responses of AP-TPO and TPO-AP; Figure S3: RTPL and LTPL (77K) in solution state AP-TPO and TPO-AP; Figure S4: Device lifetime at 1000 nits of AP-TPO and TPO-AP.
