# Supplementary material for: A High-Efficiency Deep Blue Emitter for OLEDs with a New Dual-Core Structure Incorporating ETL Characteristics
Source: Molecules. 2023 Nov 8;28(22):7485. doi: 10.3390/molecules28227485 (PMC10673098; doi:10.3390/molecules28227485)
Supplement: Supplementary file 1 [file molecules-28-07485-s001.zip › molecules-2694465-supplementary.pdf]

Supporting information

A High-Efficiency Deep Blue Emitter for OLEDs with a New Dual-Core Structure Incorporating ETL Characteristics

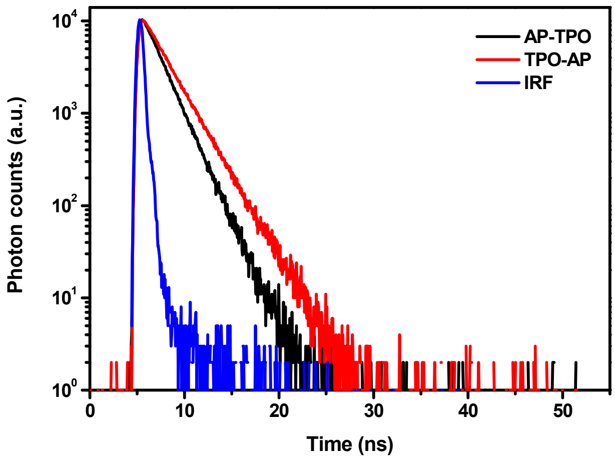

Figure S1. TRPL curves of AP-TPO and TPO-AP.

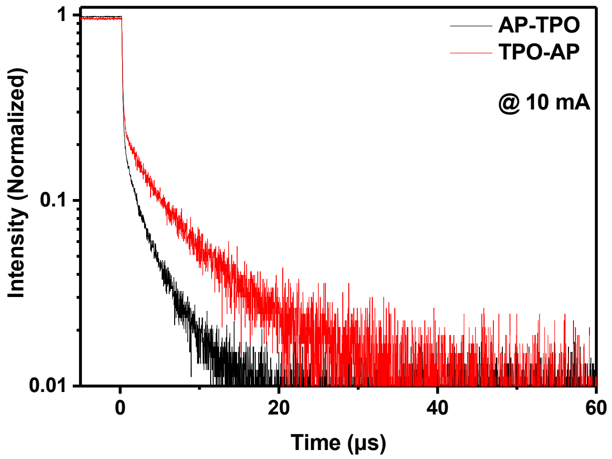

Figure S2. Transient EL responses of AP-TPO and TPO-AP.

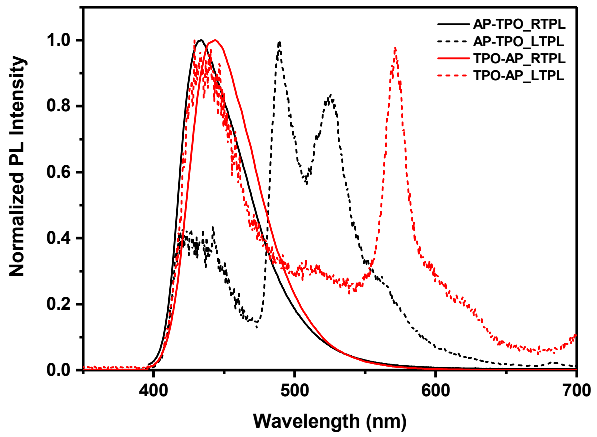

Figure S3. RTPL and LTPL (77K) in solution state AP-TPO and TPO-AP.

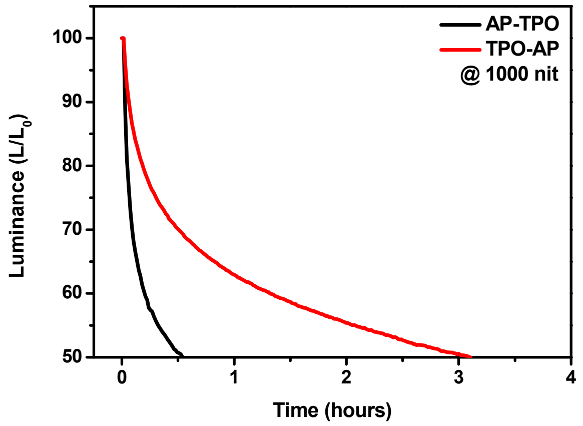

Figure S4. Device lifetime at 1000 nits of AP-TPO and TPO-AP.

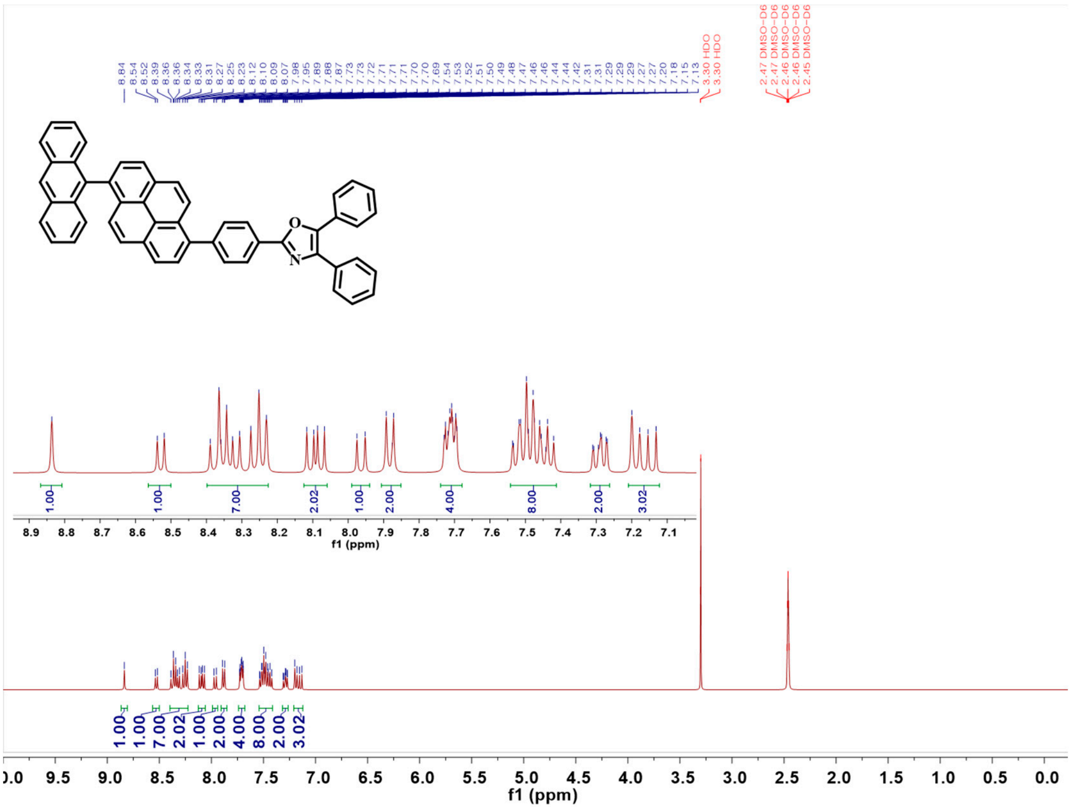

Figure S5. ^{1}H NMR spectra of compound (5) in DMSO.

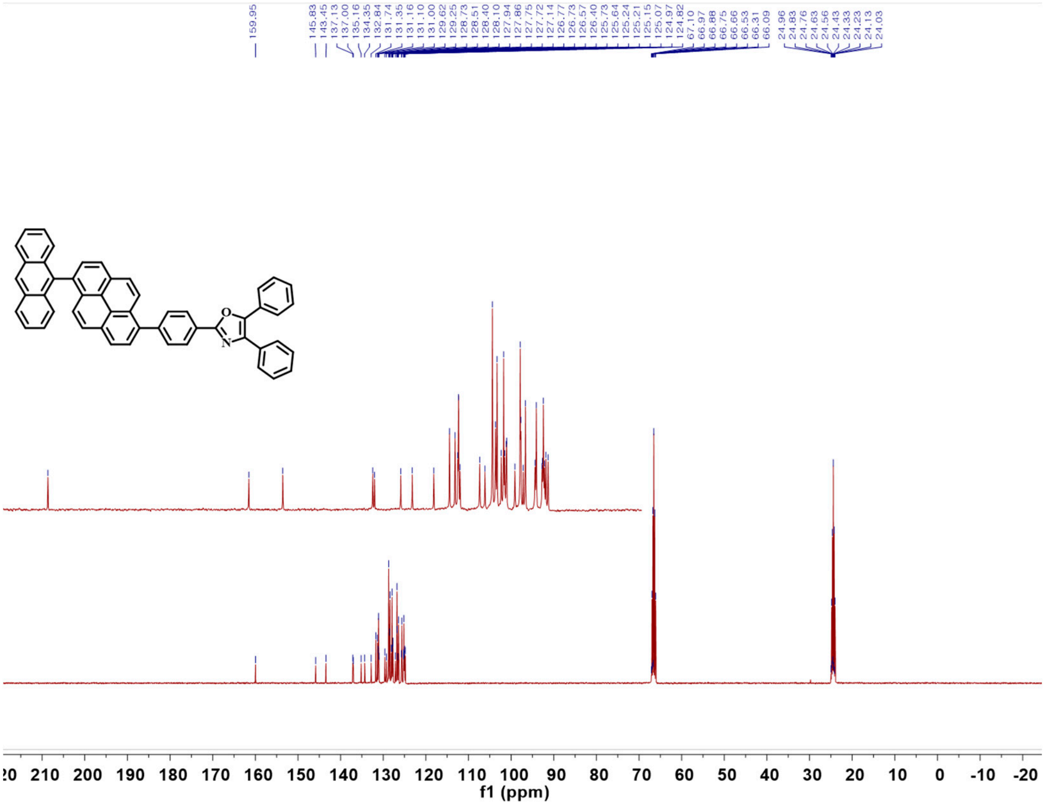

Figure S6. ^{13}C NMR spectra of compound (5) in THF.

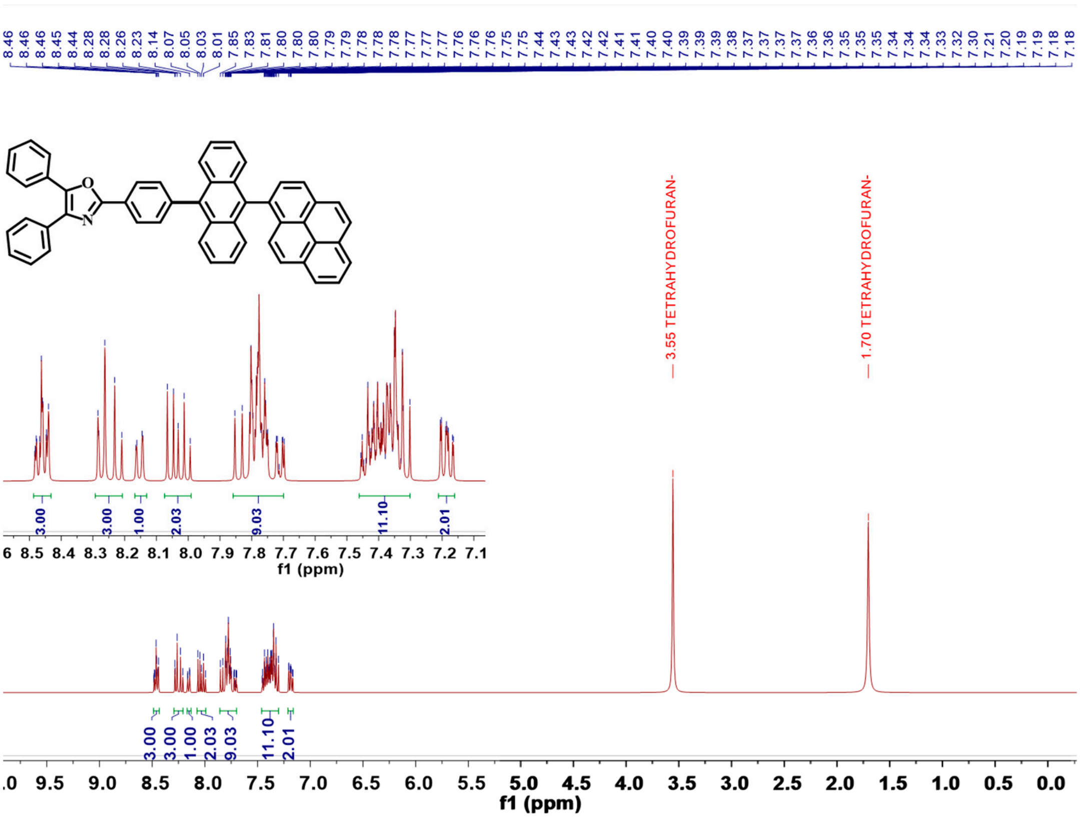

Figure S7. ^{1}H NMR spectra of compound (6) in THF.

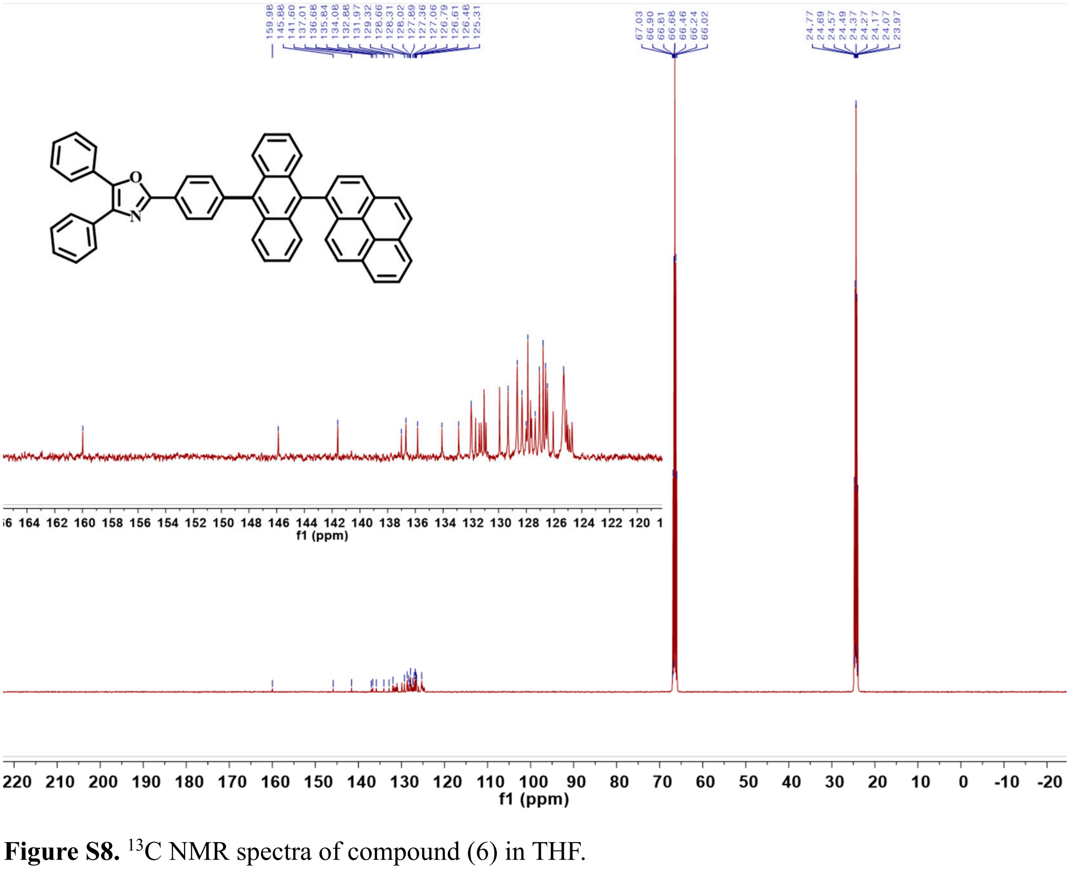

Figure S8. ^{13}C NMR spectra of compound (6) in THF.
